# Evaluating a community-based cervical cancer screening strategy in Western Kenya: a descriptive study

**DOI:** 10.1186/s12905-018-0586-0

**Published:** 2018-07-03

**Authors:** Megan Swanson, Saduma Ibrahim, Cinthia Blat, Sandra Oketch, Easter Olwanda, May Maloba, Megan J Huchko

**Affiliations:** 1 Department of Obstetrics and Gynecology, Division of Gynecologic Oncology, University of California at San Francisco, Mission Hall, 7th Floor, Room 7444, Box 0132, 550 16th St, San Francisco, CA 94158 USA; 2 Research care and Training Programme/FACES NGO, Kenya Medical Research Institute, Nairobi, Kenya; 3 Department of Obstetrics and Gynecology, UCSF, San Francisco, USA; 4 Duke Department of Obstetrics and Gynecology, Global Health Institute, Durham, USA

**Keywords:** Cervical cancer screening, Sub-Saharan Africa, Community health campaign, Self-collection

## Abstract

**Background:**

The incidence of cervical cancer in Kenya is among the highest in the world. Few Kenyan women are able to access screening, thus fueling the high cervical cancer burden. Self-collected human papilloma Virus (HPV) tests, administered during community-health campaigns in rural areas may be a way to expand access to screening.

**Methods:**

In December 2015, we carried out a four-day community health campaign (CHC) to educate participants about cervical cancer prevention and offer self-administered HPV screening. Community enumeration, outreach and mobilization preceded the CHC. Samples were sent to Migori County Hospital for HPV DNA testing using *care*HPV Test Kits. Women were notified of results through their choice of short message service (SMS), phone call, home visit or clinic visit. HPV positive women were referred for cryotherapy following a screen-and-treat strategy.

**Results:**

Door-to-door enumeration identified approximately 870 eligible women in Ngodhe Community in Migori County. Among the 267 women attending the campaign, 255 women enrolled and collected samples: 243 tests were successfully resulted and 12 were indeterminate. Of the 243 resulted tests, 47 (19%) were positive for HPV, with young age being the only significant predictor of positivity. In multivariate analysis, each additional year of age conferred about a 4% decrease in the odds of testing positive (95% CI 0.1 to 7%, *p* = 0.046). Just over three-quarters of all women (195/255), were notified of their results. Those who were unable to be reached were more likely to prefer receiving results from clinic (54/60, 90%) and were less likely to have mobile phones (24/60, 73%). Although 76% of HPV positive women were notified of their results, just half (51%) of those testing positive presented for treatment. HPV positive women who successfully accessed the treatment facility did not differ from their non-presenting counterparts by demographics, health history, desired route of notification or access to a mobile phone.

**Conclusion:**

Nearly a third of eligible women in Ngodhe Community attended the CHC and were screened for cervical cancer. Nearly all women who attended the CHC underwent cervical cancer screening by self-collected HPV tests. Three-quarters of all participants received results, but just half of HPV positive participants presented for treatment in a timely fashion, suggesting that linkage to treatment remains a major challenge.

**Trial registration:**

NCT02124252, Registered 25 April 2014.

## Background

While cervical cancer is the fourth most-common cancer among women worldwide, there is dramatic disparity in incidence globally [1]. Cervical cancer is over-represented in low- and middle-income countries (LMICs), which account for 84% of the cases and 87% of the deaths [1]. Incidence in East Africa is among the highest in the world. While incidence has decreased in developed countries secondary to widespread cytologic screening, cervical cancer rates in East Africa have increased in recent years.

Kenya experiences a high cervical cancer burden. The crude incidence rate is 22.4 per 100,000 women [2], about three times higher than the incidence rate in the United States [3]. The lack of screening programs is largely responsible for the high incidence of cervical cancer in Kenya, as in most of sub-Saharan Africa [4]. Few women in Kenya are ever screened for cervical cancer; in 2003, 3.5% of age-appropriate women reported ever-undergoing screening [2, 5]. Access to screening is limited by lapses in service availability, lack of emphasis on preventative care and perceived unacceptability of pelvic exams [6].

Cytology-based screening is not practical for wide-spread use in sub-Saharan Africa due to its high cost, low sensitivity, inherent need for a laboratories and trained technicians and complex follow-up protocols [4]. Testing for Human papillomavirus (HPV), the causative agent in almost all cervical cancer, is recommended as the primary screening modality where feasible [7, 8]. HPV DNA testing is the most objective and sensitive screening approach [9–12], and has been shown to decrease mortality from cervical cancer in low-resource settings [13]. Visual inspection with acetic acid (VIA) is an acceptable alternative where HPV testing is cost-prohibitive [7, 8]. Data suggest that self-collection of HPV, has comparable sensitivity to clinician-collection and is well-tolerated by women [11, 12, 14, 15]. A simulation model based on epidemiologic data from Uganda shows that HPV testing may be more cost-effective than VIA [16].

The impact of any successful screening program depends on widespread uptake and linkage to treatment for those who screen positive. Despite the potential for overtreatment, “screen-and-treat” approaches, ideally with HPV testing as the sole screening test or HPV followed by VIA triage, are recommended over approaches involving colposcopy and/or cytologic or histologic confirmation of high-grade dysplasia [8, 10]. When treatment requires a separate visit to a health facility, some degree of attrition is expected. Loss to follow-up can significantly reduce the impact and cost-effectiveness of a screen-and-treat program [17].

An alternative to clinic-based screening is periodic, high-volume community health campaigns (CHCs) offering self-collected HPV testing with referral for treatment when necessary. Taking screening outside of the clinic and utilizing self-collected samples overcomes the need for a pelvic exam, which Kenyan women have described as unacceptable, [6] and other barriers associated with clinic attendance and staffing. Community-based healthcare has gained traction in recent years because it can mobilize a large proportion of a community, and may be less resource-intensive than receiving similar preventive care at clinics. CHCs have been utilized for other preventive and diagnostic health services, including antenatal care, [18–20] malaria, [21] diarrheal disease, [22, 23] human immunodeficiency virus (HIV) testing and treatment, [24, 25] and tuberculosis detection. [26] An HIV prevention campaign in rural Kenya tested a community-based healthcare strategy, and found increased antiretroviral (ART) coverage with cost savings [27, 28].

We carried out a community health campaign (CHC) in Ngodhe Community in Migori County to inform a two-phase cluster randomized trial of implementation strategies for cervical cancer prevention. The aim of the present study was to assess the uptake and acceptability of the CHC and subsequent self-collected HPV test, as well as to report the HPV prevalence and proportion of screen-positive participants who successfully obtained treatment.

## Methods

In December 2015, we conducted a four-day community health campaign (CHC) offering education about cervical cancer precursors and prevention, as well as self-administered screening for HPV. This CHC was used to inform a two-phase cluster randomized trial of implementation strategies for cervical cancer prevention in Western Kenya.

The CHC took place in the Ngodhe Community of Kanyadeto sub-county, in Migori County. Ngodhe community, with a population of roughly 5590 was selected as the pilot site for the larger study as it is representative of the community size targeted for the cluster-randomized trial. Kenya Ministry of Health community health workers enumerated all women in the community aged 25–65 through door-to-door home visits prior to the campaign.

Community engagement activities and stakeholder meetings were carried out for two weeks before the CHC. Community health workers and study staff provided information in public places including markets, churches and women’s groups meetings about cervical cancer screening and described the upcoming campaigns as an opportunity to learn more about and undergo HPV-based testing. Women were also approached about the upcoming campaign through door-to-door outreach. Fliers and posters were also displayed to advertise the campaign. All women in Ngodhe aged 25–65 were invited to participate in the CHC and screening. Participation was voluntary and subjects incurred no cost.

Upon arrival at the CHC, women were screened for eligibility. Women aged 25–65 living in Ngodhe with no prior total hysterectomy or history of cervical cancer were eligible to participate. Women outside the age-range or with a history of cervical cancer or total hysterectomy for any indication were excluded. Women then participated in a group education module on cervical cancer, followed by a description of study procedures and participation. Individual informed consent was then obtained and followed by a brief survey collecting demographics, basic health information and reproductive health history. Women were then provided the testing kit, given self-collection instructions with diagrams for clarification and directed to private areas within the tent for the self-collection. In private spaces created for the campaign, participants inserted test kit brush into the vagina and placed the brush tip into a specimen cup before sealing. Sampling instructions and diagrams were also displayed in these private areas.

After collection, specimens were given to study staff, who conducted a post-test survey to assess acceptability of self-testing and to obtain phone numbers and preferred route of notification of results. Four notification options were offered: short message service (SMS), phone call, home visit, or collection of results from their nearest health facility. Study staff provided participants with their results via their preferred notification option. SMS notification was considered successful if transmission of text message was confirmed by the Frontline SMS™ program (i.e. phone was on, SIM card valid, line active). Phone and home visits were successful if the participant was reached in person, and given their results directly by study staff. Clinic notification was considered successful if the participant returned to her nearest clinic to pick up her results by clinic staff.

Specimens were transported to Migori County Hospital for processing and batch analysis with the *care*HPV Test Kit (Qiagen, Germantown, MD, USA). The laboratory was staffed by one certified laboratory technician and a research assistant with spent approximately 30% of her time there. The *care*HPV Test Kit, specifically designed for use in LMICs, is a signal-amplification test for high-risk HPV DNA detection. Antibodies bind to magnetic beads, rapidly capturing specific target HPV nucleic acid sequences, which are then detected using a chemiluminescence signal. This method can qualitatively detect 14 types of high-risk HPV (16, 18, 31, 33, 35, 39, 45, 51, 52, 56, 58, 59, 66, and 68) in cervical or vaginal specimens. More than 80 specimens can be processed in 2.5 h [29]. If samples could not be analyzed within three days of collection, they were stored in a refrigerator for up to two weeks prior to analysis.

Results were sent to the data team, who supplied them to the research assistants and community health workers, along with the standard follow-up plan. The community health workers then notified participants of their results by their preferred route. The study coordinator oversaw all the results. Follow-up guidelines were provided per Ministry of Health protocol. HIV negative women who tested negative for HPV were instructed to repeat screening in five years. If women were HIV-infected and tested HPV negative, they were told to repeat screening in one year. Women who tested positive for HPV were instructed to attend Migori County Hospital for treatment according to a screen-and-treat model.

All women who tested HPV positive were offered treatment at the hospital, with a pre-treatment visual inspection with acetic acid (VIA) to determine mode of treatment. Cryotherapy was the standard treatment for those women with an entirely visible lesion and squamocolumnar (SC) junction, with lesions covering less than 75% of the cervix, no extension to the endocervix or vagina and no evidence of cancer. According to a screen-and-treat strategy, women who had an otherwise satisfactory VIA with no identified lesions were also offered cryotherapy. Cryotherapy was performed by nurses who had specific cervical cancer screening training, including VIA and cryotherapy. Loop Electrosurgical Excision Procedure (LEEP) would have been offered to those with lesions not otherwise amenable to ablative therapy. Should LEEP have been necessary, the medical superintendent (a medical officer) would have provided it. Women with lesions highly suspicious for cancer on visual inspection were offered biopsy and referred to a Provincial Hospital for further management.

To test for association between HPV positivity and categorical explanatory variables, we performed chi-squared tests and Fisher’s exact tests. For continuous explanatory predictors, we used two independent-sample t-tests to compare sample means by outcome. Subjects for whom the outcome variable (HPV test result) was missing (test result was “indeterminate”) were compared to the other participants to evaluate potential bias. We used logistic regression to explore the association of demographic and reproductive health variables with this outcome. *P* values less than 0.05 were considered statistically significant. Factors known or theorized to be associated with HPV infection or those found to be significantly associated with high-risk HPV in the bivariate analysis were considered for multivariate analysis.

We also considered two other outcomes: successful notification of results (for all participants) and successful presentation for treatment (among HPV positive participants). We tested these two outcomes for association with demographic and health history variables. Association with categorical explanatory variables was explored with chi-squared tests and Fisher’s exact tests. For continuous explanatory predictors, we used two independent-sample t tests to compare sample means by outcome. We also used logistic regression to look at unadjusted odds ratios of successful presentation for treatment by aforementioned predictors. All data were analyzed using Stata version 14.2 (College Station, Texas, USA).

## Results

**Fig. 1:**
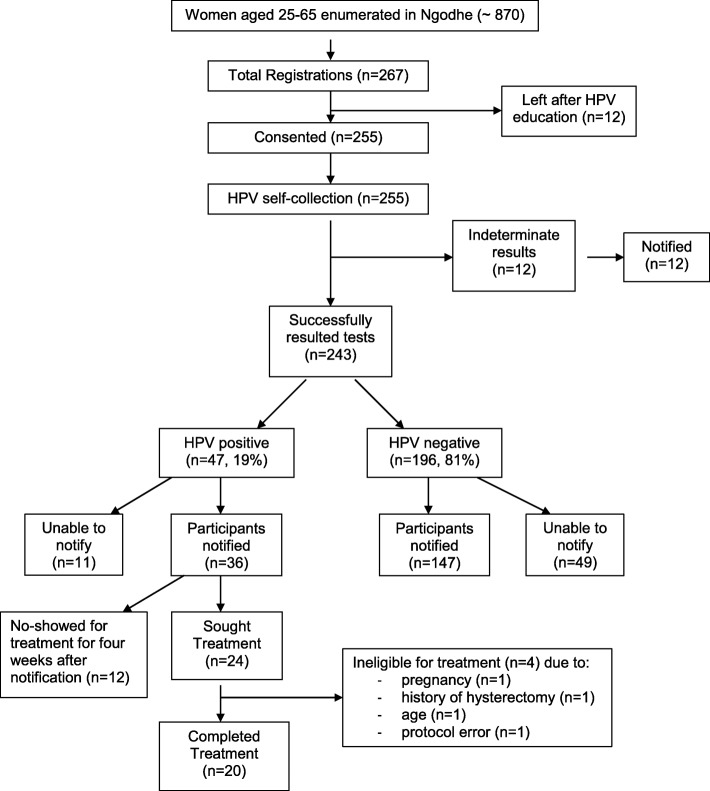
Study flow diagram

During the door-to-door enumeration, community health workers identified approximately 870 women aged 25–65 in Ngodhe Community. During the four-day campaign, 267 (31%) attended the health campaign education component. Of these, 255 consented to participate in the survey and obtain a vaginal swab for high-risk HPV testing. All 255 participants collected a sample, which was sent to the lab for processing: 243 tests were successfully resulted and 12 tests were read as “indeterminate.” The 12 women with indeterminate tests were compared to the 243 women with HPV tests yielding a result. The women with indeterminate samples were not different from the women with sufficient samples in terms of age, history of prior screening, HIV positivity, use of family planning, and/or current pregnancy. The 243 participants with valid HPV results were included in this analysis (Fig. 1).

The mean age of study participants was 36 years (SD +/− 11 years). Nearly one in five (19%) women self-reported prior cervical cancer screening: of those 63% had been screened using VIA. About one-quarter (24%) of participants self-reported as HIV positive. Family planning was used by 44% of the respondents, with the most common method being implants. Five percent of the participants were currently pregnant.

Forty-seven (19%) women had positive HPV tests. In unadjusted analysis, none of the previously described variables (age, history of prior screening, HIV serostatus, use of family planning, nor current pregnancy) differed by HPV result (Table 1).

**Table 1:** Characteristics of participants in a cervical cancer screening Community Health Campaign by HPV status

Variable	Total N	HPV negative %	HPV positive %	Unadjusted Odds Ratio of HPV positivity (95% CI)	Adjusted^a^ Odds Ratio of HPV positivity (95% CI)
Total (N, %)	*N* = 243	*N* = 196 (81%)	*N* = 47 (19%)		
Age (mean, SD)	36 (11)	37 (11)	34 (11)	0.97 (0.94–1.01)	0.96 (0.93–0.99)
Age: categorical					
25–29 yo	97	75	25	1.41 (0.55–3.62)	^b^
30–39 yo	67	82	18	0.94 (0.33–2.63)	^b^
40–49 yo	42	90	10	0.45 (0.12–1.69)	^b^
≥ 50 yo	37	81	19	1.0	^b^
Prior screening history					
None prior	197	81	19	1.0	1.0
Prior screening	46	80	20	1.02 (0.45–2.29)	0.98 (0.41–2.34)
Prior screening type					
No prior screening	197	81	19	1.0	^b^
VIA	29	79	21	1.09 (0.42–2.87)	^b^
Pap smear	15	86	14	1.05 (0.28–3.89)	^b^
HPV test	1	100	0	too few observations	^b^
HIV serostatus					
HIV -	178	81	19	1.0	1.0
HIV +	57	79	21	1.13 (0.54–2.36)	1.29 (0.59–2.83)
Family Planning Method					
No method	137	79	21	1.0	1.0
Modern method	106	83	17	0.76 (0.40–1.46)	0.53 (0.25–1.12)
Family Planning					
No method	137	79	21	1.0	^b^
Implant	45	76	24	omitted for collinearity	^b^
Injectable	39	90	10	0.43 (0.14–1.29)	^b^
Other	22	86	14	0.59 (0.16–2.13)	^b^
Pregnant					
Not pregnant	216	81	19	1.0	1.0
Pregnant	12	75	25	1.38 (0.36–5.32)	0.84 (0.20–3.50)

^a^Adjusted for age, history of previous screening, HIV serostatus, use of a family planning method, and pregnancy

^b^not included in the multivariate model

In multivariate logistic regression model of HPV positivity (adjusted for age, history of previous screening, HIV serostatus, use of a family planning method and pregnancy), each additional year of age conferred about a 4% decrease in the odds of testing positive (95% CI 0 to 7%, *p* = 0.034). No other predictor variable (a history of prior screening, HIV positivity, use of family planning, or pregnancy) was independently associated with HPV-positivity (Table 1).

Women’s experience with self-collection was overall positive. All of the 255 participants who obtained a vaginal swab said the self-sampling instructions were clear and almost all (98%) felt they had adequate privacy. Despite 16% of participants reporting that the test caused some pain, nearly all (98%) said they would test again via self-collection, and 99% would recommend the procedure to a friend.

Women who completed screening indicated their preferred route of results notification. Based on eventual HPV results, 67% of participants would have wished to receive these results by text, 4% preferred a phone call, 5% wanted a home visit and 24% wished to pick up their results from a clinic. The women who were unable to be reached to deliver HPV results were less likely to have access to a mobile phone (40% said they did not have a phone, compared to 5% of the women who were notified, chi2 *p* < .001). Otherwise, women reached with results were not different from those unreachable in terms of age, HIV status, history of prior cervical cancer screening, use of a family planning method, current pregnancy or HPV test results (Table 2). The mean and median number of days from testing to receiving results was 28 days.

**Table 2:** Characteristics of participants by whether or not they were successfully notified of HPV test results

Variable	Total N	Unable to be reached, not notified of results %	Successfully notified of results %	*P* value (t-test, chi-square or Fisher’s exact test)
Total (N, %)	*N* = 255	*N* = 60 (24%)	*N* = 195 (76%)	
Age (mean, SD)	36 (11)	38 (13)	35(11)	0.152
Age: categorical				
25–29 yo	104	21	79	0.629
30–39 yo	71	23	77	
40–49 yo	42	24	76	
> = 50 yo	38	32	68	
Prior Screening History				
None prior	208	25	75	0.122
Prior screening	47	15	85	
HIV serostatus				
HIV-	189	23	77	0.891
HIV+	58	22	78	
Family Planning				
No method	142	24	76	0.861
Using a method	113	23	77	
Pregnant				
Not pregnant	227	23	77	0.196
Currently pregnant	13	38	62	
Have access to a mobile phone				
No access	33	73	27	< 0.001
Has phone	222	16	84	
Desired Method of Notification of test results via:				
Text	171	0	100	< 0.001
Phone call	11	36	64	
Home visit	12	17	83	
Clinic visit	61	89	11	
HPV test results				
HPV positive	47	23	77	0.141
HPV negative	196	25	75	
Indeterminate	12	0	100	

Twenty-four (51%) of the 47 HPV-positive women sought treatment within 4 weeks of receiving their results, with the median time interval between notification of results and presentation for treatment being 7 days (interquartile range: 4–15). Twenty (43%) were able to successfully obtain treatment on this first visit. Those who did not complete treatment were either pregnant (*N* = 1), incorrectly not offered treatment for lack of visible lesion (*N* = 1), and two were found to be ineligible for the study secondary to reported-age (*N* = 1) or a history of a hysterectomy (*N* = 1). All the HPV positive women treated were appropriate candidates for ablation and underwent cryotherapy.

There was no difference between HPV positive women who accessed treatment and those who did not with respect to age, HIV status, history of prior cervical cancer screening, use of a family planning method or current pregnancy. The methods chosen for results notification did not differ significantly and neither group was more or less likely to have access to a mobile phone (Table 3).

**Table 3:** Characteristics of HPV + participants by whether or not they successfully presented for treatment

Variable	Total N	Unable to present for treatment (no-show) %	Presented for treatment %	Unadjusted Odds Ratio of Presenting for Treatment (95% CI)
Total (N, %)	*N* = 46	*N* = 23 (49%)	*N* = 24 (51%)	
Age (mean, SD)	34 (12)	31 (11)	36 (12)	1.04 (0.99–1.10)
Age: categorical				
25–29 yo	24	58	42	0.29 (0.05–1.78)
30–39 yo	12	50	50	0. 40 (0.05–2.93)
40–49 yo	4	25	75	1.20 (0.07–19.63)
> = 50 yo	7	29	71	1.0
Prior Screening HIstory				
None prior	38	50	50	1.0
Prior screening	9	44	56	1.25 (0.29–5.39)
HIV serostatus				
HIV-	34	47	53	1.0
HIV+	12	50	50	0.89 (0.24–3.32)
Family Planning				
No method	29	48	52	1.0
Using a method	18	50	50	0.93 (0.29–3.03)
Pregnant				
Not pregnant	42	48	52	1.0
Currently pregnant	3	100	0	too few observations
Have access to a mobile phone				
No access	8	63	37	1.0
Has phone	39	46	54	1.94 (0.41–9.29)
Notification of test results via:				
Text	17	53	47	1.0
Phone call	11	55	45	0.94 (0.20–4.29)
Home visit	12	25	75	3.38 (0.67–17.00)
Clinic visit	7	71	29	0.45 (0.34–2.30)

## Discussion

This is the first study that has evaluated the use of a CHC to offer HPV-testing and linkage to treatment in Kenya. The integration of a CHC into a cervical cancer screening program was successful in many respects. Nearly a third of the population in Ngodhe was screened during the four-day campaign, a vast improvement over the baseline reported national screening rate of 3% [5]. A relative strength of this screening model was the ability to notify three-quarters of participants regarding test results by their preferred communication method. A study in Uganda utilizing community-based self-collection for HPV testing reported reaching just 47% of their HPV positive participants for results notification via phone calls [30].

Although just over half of HPV positive women accessed treatment in a timely fashion. For these women, the model worked efficiently: the test itself was overall well-tolerated, they received results according to the method of their choosing and were treated within seven days of notification. The experience of these women provides a basis by which we can work to improve and strengthen the model to serve more women.

Cost effectiveness analyses have demonstrated screen-and-treat strategies using HPV testing, even once-per-lifetime screening models, to be the most effective at decreasing lifetime risk of cancer and potentially the most cost-effective. However, effectiveness wanes as loss-to-follow-up increases [16, 31]. In a simulation model based on epidemiologic data from Uganda, when loss-to-follow-up approached 40%, two-visit HPV testing and treatment was still generally more effective than one-visit VIA, but was no longer the more cost-effective method, as it was, compared to VIA, when loss-to-follow-up was modeled at 10%. At 60% loss-to-follow-up, one-visit VIA, even with it’s lower sensitivity, actually became more effective at decreasing lifetime risk of cervical cancer [16].

Loss-to-follow-up decreases the effectiveness of any cervical cancer prevention cascade. Loss-to-follow-up in cervical cancer screening cascades in LMICs using various methodologies has been reported to range between 17 and 45%, with most studies reporting at least one-third [4, 32–37]. Our loss-to-follow-up of 49% does not substantially deviate from that of the reported literature, but does suggest a need for strengthening linkage strategies.

While the women who were able to access treatment were not significantly different than their HPV positive counterparts who did not present, receiving results by home visit was associated with higher odds (though not significant) and collecting results at a nearby clinic was associated with lower odds of accessing the treatment facility compared to receiving results by text message (the most commonly preferred route). Moreover, almost all of the 60 women who were not even reached to deliver test results (90%), had elected to pick up their results in a nearby clinic. These women were also significantly less likely to have access to mobile phones. These trends suggest that more resource- and personnel-intensive home visits, maybe be a better platform for delivering results in this screen-and-treat model.

Decreasing loss to follow-up can be accomplished through fewer visits or improved linkage to treatment. One-day screen-and-treat visits using the *care*HPV test kits are theoretically possible, but samples are usually batched, allowing for quick, but not point-of-care immediate results. Running the test assay would not have been feasible at our community-based screening sites. Thus, improving linkage to treatment is the only way to improve adherence in a two-step screen-and-treat model. Minimizing the time from test to receipt of results will be an important consideration as this model is scaled up. Home visits by Community Health Workers have been shown to improve adherence to scheduled follow-up visits in cytology-based cervical cancer screening programs in South Africa [17, 38]. Home visit, as a method for results delivery, was associated with higher odds of accessing treatment, though not significant, in this study; suggesting that including home-visits by community health workers may be an effective component of a linkage intervention. However, access to mobile phones was also key for successfully notifying participants of their results: texting was the most popular route of results delivery and not having a phone was associated with inability to reach with test results. The success of text notification of results provided preliminary justification for the larger trial to include this option as part of the larger implementation strategy.

A limitation of this study was our sample size. While the reported prevalence of HPV (19%) is similar to that reported by other studies from the region, [30, 36, 39] this left us with only 47 HPV positive women needing further treatment. With such a small number of HPV positive women, we are limited in our ability to detect predictors of successfully accessing treatment. In addition, as this was an implementation rather than a clinical trial, we were only able to look at HPV and not cervical intraepithelial neoplasia as an outcome. There were also limitations associated with obtaining results including the number of tests that resulted as “indeterminate” and the turn-around time resulting in a mean/median 28 days from test to results delivery. Investigators successfully addressed the indeterminate finding with *care*HPV support staff and the Migori-based laboratory. Investigators are also striving to minimize delays in results and notification in preparation for the planned two-phase cluster randomized trial of implementation strategies for cervical cancer prevention in Western Kenya.

## Conclusions

This study evaluating a CHC as the planning step in a cervical cancer screen-and-treat strategy shows that high-volume, short-duration campaigns can be a successful way to increase screening and to successfully deliver results to participants. However, observed follow-up rates in this study of just 51% suggest the need for improved linkage to treatment. Extrapolating from data from Uganda, loss to follow-up at this level likely indicates that HPV testing is still probably more effective than VIA in decreasing lifetime risk of cancer, but at a much higher cost than VIA. Decreasing loss to follow-up could bridge the cost disparity. Given trends in our data and findings from other studies, [17, 38] home visits by community health workers, possibly as a standard route of results notification or in addition to results notification, may be a strategy to improve adherence. Different linkage strategies should be tested to see if loss to follow up can be decreased, thereby increasing both effectiveness and cost-effectiveness of HPV testing in screen-and-treat models in LMICs.

## Abbreviations

ARV: Antiretroviral
CHC: Community health campaign
CI: Confidence interval
HIV: Human immunodeficiency virus
HPV: Human papillomavirus
IRB: Institutional review board
KEMRI: Kenya Medical Research Institute
LEEP: Loop electrosurgical excision procedure
LMICs: Low- and middle-income countries
SC: Squamocolumnar
SERU: Scientific and ethical review unit
SIM: Subscriber identity module
SMS: Short message service
VIA: Visual inspection with acetic acid
